# Relationship of p73 gene polymorphism and additional gene-smoking and gene-obesity interaction with non-small cell lung cancer risk

**DOI:** 10.18632/oncotarget.16257

**Published:** 2017-03-16

**Authors:** Qiuge Wu, Yan Shi, Lulu Ge, Dongbo Ma, Hui Zhang, Jing Wang

**Affiliations:** ^1^ Department of Respiratory and Critical Care Medicine, The First Affiliated Hospital of Zhengzhou University, Henan, China

**Keywords:** non-small cell lung cancer, G4C14-to-A4T14, polymorphism, interaction, smoking

## Abstract

**Aim:**

The aim of this study was to investigate the impact of G4C14-to-A4T14 polymorphism within P73 gene and additional interactions with current smoking and obesity on non-small cell lung cancer (NSCLC) risk in a Chinese population.

**Results:**

Logistic regression analysis showed a significant association between genotypes of the AT allele in G4C14-to-A4T14 and decreased NSCLC risk. NSCLC risk was significantly lower in carriers of the G4C14-to-A4T14- AT allele than those with GC/GC genotype (AT/AT + GC/AT versus GC/GC), adjusted OR (95%CI) = 0.68 (0.55–0.93). We also found that the OR (95%CI) was 1.88 (1.32-2.47) for current smokers compared with never smokers and 0.69 (0.40–0.95) for obese subjects compared to participants with normal BMI. Never smokers with AT/AT or GC/AT of the G4C14-to-A4T14 genotype have the lowest NSCLC risk compared with smokers with the GC/GC genotype after covariates adjustment, OR (95%CI) = 0.52 (0.26-0.87). Obese participants with G4C14-to-A4T14- AT/AT or GC/AT genotype have the lowest NSCLC risk compared with non- obese subjects with the GC/GC genotype after adjusting for covariates, OR (95% CI) = 0.56 (0.33–0.85).

**Materials and Methods:**

A logistic regression model was used to examine the association between G4C14-to-A4T14 polymorphism and NSCLC, and its interaction with current smoking and obesity. The odds ratios (OR) and 95% confident intervals (95%CI) were calculated.

**Conclusions:**

Our results support an important association between the G4C14-to-A4T14 and decreased NSCLC risk and additional impact of an interaction between G4C14-to-A4T14 and smoking or obesity on NSCLC risk.

## INTRODUCTION

Non-small cell lung cancer (NSCLC) was a subtype for lung cancer (LC), and was the main cause of cancer-related deaths around the world [1, 2]. The NSCLC incidence was largely determined by tobacco smoking. Recent years, LC rates have been increasing rapidly in China, where the smoking rates were also increased rapidly [2]. However, not all lung cancer cases have the smoking history [3, 4]. Therefore, genetic factors were considered as other important risk factors for lung cancer, and LC susceptibility could be influenced by both environmental and genetic factors, and their gene-environment interactions. Recently, epidemiologic studies [5–7] also indicated that overall obesity, as measured by body mass index (BMI) was associated with a reduced susceptibility to lung cancer.

The p73 gene was located at human chromosome 1p36.33 [8, 9]. It has been suggested that commonly loss of heterozygosity (LOH) existed in many subtypes of cancer [8, 10, 11], and LOH rate at the p73 gene was high (62%) among lung cancer patients [12]. Recently, a kind of polymorphism involves two single-nucleotide polymorphisms (SNPs) at positions 4 (G-A) and 14 (C-T) (G4C14-to-A4T14) were more reported [8]. Several previous studies have indicated the association between G4C14-to-A4T14 and NSCLC risk in different populations [13–15]; however, they concluded inconsistent results. In addition, more and more evidence suggested that lung cancer was significantly associated with both genetic factor and environmental factors in the general population. Smoking have an important role in NSCLC, and two studies [16, 17] have focused on the interaction between P73 gene and some environmental factors on lung cancer risk, but they obtained conflicting results. No study focused on the interaction between P73 gene and obesity, which was also an important risk factor for lung cancer [5–7]. Thus, the aim of this study was to investigate the impact of G4C14-to-A4T14 polymorphism and additional interactions with current smoking and obesity on NSCLC risk in a Chinese population.

## RESULTS

A total of 1382 participants with a mean age of 68.1 ± 13.9 years were selected, including 764 males and 618 females, consist of 460 NSCLC cases and 922 controls. Table 1 shows the general characteristics for cases and controls. The distributions for obesity, both smoking and drinking, current smokers were significantly differed between the cases and controls. The mean of BMI was lower in the NSCLC cases than the controls.

**Table 1:** General characteristic of study participants in cases and controls

Variables	NSCLC Cases (*n* = 460)	Controls (*n* = 922)	*p*-values
Age (year)	68.7 ± 15.8	67.2 ± 14.6	0.080
Males *N* (%)	246 (53.5)	518 (56.2)	0.371
Alcohol consumption *N* (%)	166 (36.1)	326 (35.4)	0.836
Current smokers *N* (%)	183 (39.8)	214 (23.2)	< 0.001
Both smoking and drinking *N* (%)	138 (30.0)	161 (17.5)	< 0.001
BMI (kg/m^2^)	23.4 ± 8.9	24.7 ± 8.6	0.009
Obesity *N* (%)	123 (26.7)	338 (36.6)	< 0.001
WC (cm)	81.6 ± 14.3	82.4 ± 14.8	0.338

Note: Means ± standard deviation for age, WC and BMI. Abbreviations: WC, waist circumference; BMI, body mass index.

No significant difference in genotype frequencies from the Hardy–Weinberg equilibrium test was noted for any tested SNPs in the controls (*P* = 0.691). The frequency for the GC/ GC allele of G4C14-to-A4T14 was lower in the NSCLC cases than that in controls. The genotypes with the AT allele in G4C14-to-A4T14 were associated with decreased NSCLC risk after adjusting for age, gender, current smoking, drinking and BMI. NSCLC risk was significantly lower in carriers of G4C14-to-A4T14- AT allele than those with GC/GC genotype (AT/AT + GC/AT versus GC/GC), adjusted OR (95%CI) = 0.68 (0.55–0.93) (Table 2). We also found that the OR (95%CI) was 1.88 (1.32–2.47) for current smokers compared with never smokers and 0.69 (0.40–0.95) for obese subjects compared to participants with normal BMI (Table 3).

**Table 2:** Genotype and allele frequencies for p73 G4C14-to-A4T14 polymorphism in cases and controls

Genotypes and Alleles	Frequencies N (%)		*OR (95%CI)^a^*	*P*-values for genotype and allele frequencies	*HWE* test
	NSCLC Cases (*n* = 460)	Controls (*n* = 922)			
Additive					
GC/GC	294 (63.9)	490 (53.1)	1.00	< 0.001	0.691
GC/AT	149 (32.4)	361 (39.2)	0.71 (0.58–0.92)		
AT/AT	17 (3.7)	71 (7.7)	0.62 (0.42–0.96)		
Dominant					
GC/GC	294 (63.9)	490 (53.1)	1.00		
AT/AT + GC/AT	166 (36.1)	432 (46.8)	0.68 (0.55–0.93)	< 0.001	
Recessive					
GC/GC+ GC/AT	443 (96.3)	851 (92.3)	1.00		
AT/AT	17 (3.7)	71 (7.7)	0.62 (0.38–1.03)	0.004	
Allele					
GC	737 (80.1)	1341 (72.7)		< 0.001	
AT	183 (19.9)	503 (27.3)			

^a^ Adjusted for gender, age, current smoking, drinking and BMI.

**Table 3:** the association between smoking and obesity and NSCLC risk

Genotypes and Alleles	Frequencies *N*(%)		*OR (95%CI)*	*P*-values
	Cases (*n* = 460)	Controls (*n* = 922)		
**Smoking**				
Never	277 (60.2)	708 (76.8)	1.00	< 0.001^a^
Currently	183 (39.8)	214 (23.2)	1.88 (1.32–2.47)	
**Obesity**				
No	337 (73.3)	584 (63.3)	1.00	0.020^b^
Yes	123 (26.7)	338 (36.6)	0.69 (0.40–0.95)	

^a^ Adjusted for gender, age, drinking and BMI.

^b^Adjusted for gender, age, drinking and smoking.

Stratified logistic regression analysis was used to detect the interaction between G4C14-to-A4T14 with smoking and obesity. We found that never smokers with AT/ AT or GC/ AT of the G4C14-to-A4T14 genotype have the lowest NSCLC risk compared with smokers with the GC/ GC genotype after covariate adjustment, OR (95%CI) = 0.52 (0.26–0.87) (Table 4). We also found that obese participants with G4C14-to-A4T14- AT/AT or GC/AT genotype have the lowest NSCLC risk compared with non- obese subjects with the GC/GC genotype after adjusting for covariates, OR (95%CI) = 0.56 (0.33–0.85) (Table 5).

**Table 4:** Interaction between p73 G4C14-to-A4T14 and smoking on ESCC risk

G4C14-to-A4T14	Smoking	OR (95% CI)^a^	*P*-values
GC/GC	Currently	1.00	-
GC/GC	Never	0.69 (0.48–0.94)	0.032
AT/AT + GC/AT	Currently	0.75 (0.57–1.03)	0.081
AT/AT + GC/AT	Never	0.52 (0.26–0.87)	< 0.001

^a^ Adjusted for gender, age, drinking and BMI.

**Table 5:** Interaction between p73 G4C14-to-A4T14 and obesity on ESCC risk

G4C14-to-A4T14	Obesity	OR (95% CI)^a^	*P*-values
GC/GC	No	1.00	-
GC/GC	Yes	0.64 (0.42–0.92)	0.020
AT/AT + GC/AT	No	0.72 (0.48–0.95)	0.012
AT/AT + GC/AT	Yes	0.56 (0.33–0.85)	< 0.001

^a^Adjusted for gender, age, drinking and smoking.

## DISCUSSION

In this study, genotypes of the AT allele in G4C14-to-A4T14 was associated with decreased NSCLC risk in this Chinese population. NSCLC susceptibility was lower in carriers with the AT allele of the G4C14-to-A4T14 polymorphism than those with GC/GC genotype after adjustment. Although P73 mutation was rare in several diseases, LOH at P73 locus was relatively common in lung cancer [18]. As we all known that two common SNPs have been identified to be in complete linkage disequilibrium with each other as one variation [8]. Studies have reported a direct effect of G4C14-to-A4T14 on cancer progression, particular for NSCLC risk [19, 20]. Although P73 G4C14-to-A4T14 polymorphism and its relationship with many types of cancer susceptibility have been reported previously [21, 22], few studies focused on impact of this polymorphism on NSCLC risk and the results obtained from previous studies were inconsistent. Choi et al. [23] suggested no association between the p73 G4C14-to-A4T14 polymorphism and LC risk in a Korean population. Hu et al. [24] reported that polymorphism of p73 gene was associated with a reduced LC risk in a Chinese population. However, Li et al. [16] suggested that the persons with GC/AT and AT/AT genotypes have higher LC risk. Up to now, we found limited human population study investigating the relationship between G4C14-to-A4T14 polymorphism and NSCLC risk. Wang et al. [13] indicated that the distribution for G4C14-to-A4T14 genotype was significantly different between NSCLC cases and controls. The sample in this study was relative small, so the results obtained from this study should be checked in other studies with larger sample. Wang et al. [14] also conducted another study and found that the p73 G4C14-A4T14 polymorphism and susceptibility was correlated with LC risk, but the sample size of this study was relatively small, so this association also needs further research. Another study [25] suggested that p73 may play an important role in regulating the cellular response of NSCLC to chemotherapy. Another study by Liu et al. [26] suggested that P73 G4C14-to-A4T14 polymorphisms may serve as promising biomarkers for individualized chemotherapy and prognosis of NSCLC patients.

Tobacco smoking has been widely reported important environmental risk factors for NSCLC. A recent study [27] reported that 88% of the cases of non-smokers with lung cancer were females. The NSCLC incidence was largely determined by tobacco smoking. Recent years, LC rates have been increasing rapidly in China, where the smoking rates were also increased rapidly [2]. Obesity was another modifiable risk factor for some types of cancer, including colon cancer, postmenopausal breast cancer, and so on [28]. This relationship was still inconclusive for lung cancer. In this study, we found that the smoking rate was higher in cases than that in controls; in contrast, the obesity rate was higher in controls than that in cases. In this study, we also investigated the impact of gene- smoking and gene- obesity interaction on NSCLC risk, and we found that NSCLC risk was the lowest in never smokers with AT/AT or GC/AT of the G4C14-to-A4T14 genotype, and the highest in smokers with the GC/GC genotype. In addition, NSCLC risk was also the lowest in obese participants with AT/AT or GC/AT of the G4C14-to-A4T14 genotype, and the highest in non- obese subjects with the GC/GC genotype. Two previous studies have focused on the impact of interaction between P73 gene and environmental risk factors on NSCLC risk, such as gender and age [16] and smoking [17]. Li et al. [16] suggested that the lung cancer risk associated with the AT allele (GC/AT+AT/AT) were more pronounced in younger (</= 50 years) individuals, men, and light smokers. Hiraki et al. [17] have observed the P73 gene- heavy smoking interaction on LC risk, but they concluded negative results. But in this study, we verified a significant interaction of G4C14-to-A4T14 gene- smoking and obesity interaction on NSCLC risk.

The limitations of this study should be considered. Firstly, the smoking and drinking rates in females were very low, so we could not performed sex difference analysis on this association. Secondly, the gene-gene interaction with other SNPs of P73 or other genes should be investigated in future studies. Thirdly, more environmental factors should be included in gene- environment interaction studies. Lastly, it could not omit the fact that weight loss maybe an expected side effect in lung cancer, we could not verify the time sequence for obesity and NSCLC occurrence, so this interaction effect should be verified by cohort study in the future.

In conclusion, we found that P73 G4C14-to-A4T14 polymorphism was significantly associated with NSCLC risk in Chinese population, genotypes of the AT allele in G4C14-to-A4T14 was associated with decreased NSCLC risk. We also found that never smokers with AT/AT or GC/AT of the G4C14-to-A4T14 genotype have the lowest NSCLC risk compared with smokers with the GC/GC genotype after covariate adjustment. In addition, obese participants with AT/ AT or GC/ AT of the G4C14-to-A4T14 genotype have the lowest NSCLC risk compared with non- obese subjects with the GC/GC genotype after adjusting for covariates.

## MATERIALS AND METHODS

### Subjects

This was a case-controlled study on association of G4C14-to-A4T14 polymorphism and gene-environment interaction with NSCLC genetic susceptibility. Chinese patients with NSCLC were consecutively recruited between June 2010 and July 2014 from the First Affiliated Hospital of Zhengzhou University. Patients were diagnosed and sample histology was reviewed in this hospital according to the World Health Organization tumor classification criteria. A total of 460 NSCLC patients were included in the study, controls were matched by sex, age and ethnic background and normal controls with family history of NSCLC were excluded. Blood samples were collected from each participant. Detailed personal information on demographic characteristics, smoking and drinking status were collected via interview. At recruitment, written informed consent was obtained from each participant. We defined current alcohol consumption as more than 1 drink of any type per month or not currently drinking as less than 1 drink of any type per month; current smokers were defined as those who have smoked at least 100 cigarettes and still smoked at the time of the interview; individuals with no history of cigarette smoking were considered to be never smokers. Body weight, height, and waist circumference (WC) were also measured according to standardised procedures. BMI was calculated as weight in kilograms divided by the square of the height in metres.

### Genomic DNA extraction and genotyping

Approximately 2 ml of whole blood were collected from all participants in sterile EDTA-coated vacutainers. Genomic DNA from participants was extracted from EDTA-treated whole blood using the DNA Blood Mini Kit (Qiagen, Hilden, Germany) according to the manufacturer's instructions and stored at −80°C until used for genotyping. Genotyping for G4C14-to-A4T14 within P73 gene was performed using the TaqMan platform produced by the BGI Company, China. Genotyping was repeated on a 10% random sample of study participants, and the results were 100% concordant. A 25-μl reaction mixture included 1.25 μl SNP Genotyping Assays (20×), 12.5 μl Genotyping Master Mix (2×), 20 ng DNA, and conditions as follows: initial denaturation for 10 min and 95°C, denaturation for 15 s and 92°C, and annealing and extension for 90 s and 60°C (50 cycles).

### Statistical analysis

The means and standard deviations (SD) were calculated for normally distributed continuous variables, and the *t* test was used for comparison between cases and controls; percentages were calculated for categorical variables, and the *χ*^2^ test was used for comparison between the case and control group (version 19.0; SPSS Inc., Chicago, IL). Hardy-Weinberg equilibrium (HWE) was performed using SNPStats (available online at: http://bioinfo.iconcologia.net/SNPstats). A logistic regression model performed by SPSS package (version 19.0; SPSS Inc., Chicago, IL) was used to examine the association between G4C14-to-A4T14 and NSCLC and the interaction between G4C14-to-A4T14 and smoking or obesity. The odds ratios (OR) and 95% confident intervals (95%CI) were calculated. ORs were adjusted for gender, age, smoking and BMI.
